# Quercetin Derivatives from *Bidens pilosa* Suppressed Cell Proliferation via Inhibition of RSK2 Kinase and Aldose Reductase Enzymes: UPLC-MS/MS, GC–MS, In Vitro, and Computational Studies

**DOI:** 10.1007/s12010-024-05134-8

**Published:** 2025-01-06

**Authors:** Doaa S. Ali, Alaadin E. El-Haddad, Hussein S. Mohamed, Ashraf A. El-Bassuony, Momtaz M. Hegab, Gehad AbdElgayed, Hossam Ebaid, Shimaa A. Ahmed, Emadeldin M. Kamel

**Affiliations:** 1 Chemistry of Medicinal and Aromatic Plants Department, Research Institute of Medicinal and Aromatic Plants, Beni-Suef University, Beni-Suef, 62514 Egypt; 2 Pharmacognosy Department, Faculty of Pharmacy, October 6 University, Giza, Egypt; 3 Chemistry Department, Faculty of Science, Beni-Suef University, Beni-Suef, 62514 Egypt; 4 Botany and Microbiology Department, Faculty of Science, Beni-Suef University, Beni-Suef, Egypt; 5 Integrated Molecular Plant Physiology Research, Department of Biology, University of Antwerp, 2020 Antwerp, Belgium; 6 Department of Zoology, College of Science, King Saud University, P.O. Bo2455, 11451 Riyadh, Saudi Arabia

**Keywords:** *Bidens pilosa*, Antiproliferative, Docking, Flavonoids, GC–MS, LC–MS/MS

## Abstract

Traditionally, *Bidens* *pilosa* L. is an edible herb utilized for various ailments. The study accomplished a complete analysis of *B. pilosa* extract including UPLC/T-TOF–MS/MS, GC–MS, and in vitro antiproliferative activity, in addition to molecular docking on kinase and aldose reductase enzymes. From GC–MS analysis, the percentage of identified unsaturated fatty acids (FAs) (11.38%) was greater than saturated FAs (8.69%), while the sterols percent (39.92%) was higher than the hydrocarbons percent (6.6%). Oleic and palmitic acids are the major FAs (9.48% and 6.14%, respectively). Phytochemical profile uncovered the presence of quercetin, kaempferol, myricetin, and isorhamnetin aglycones and/or glycoside derivatives alongside apigenin, acacetin, and luteolin derivatives. *B. pilosa* extract suppressed cell proliferation in a concentration-dependent manner against SNB-19 and SK-MEL-5 cell lines (IC_50_ 1.66 ± 0.06 and 4.04 ± 0.14 mg/mL, respectively). These potentials aligned with the molecular docking results on aldose reductase and kinase enzymes with promising binding affinities (− 5.3 to − 8.89 kcal mol^−1^). *B. pilosa* metabolites were found as kinases and aldose reductase inhibitors, which rationalize their antiproliferative activity. Unfortunately, toxicity assessments were not performed to assess the safety of *B. pilosa* extract. Assessment of the therapeutic efficiency via in vivo and clinical studies is required.

## Introduction

The *Bidens* genus (Asteraceae) comprises approximately 280 species [1]. *Bidens pilosa* L. is an annual and ruderal herb that grows in tropical and subtropical regions because of its outstanding resistance to unfavorable environmental circumstances [2]. *B. pilosa* grows with numerous ridged branches (1.5–2 m). Leaves are petioled oppositely pinnate arranged, with hairy serrately ovate leaflets (3–5) [3]. *B. pilosa* has capitulum inflorescence with white ray petals and yellow centers [3]. The seeds are brown to black and widely spread by wind, adhering to clothes and animal hair allowing its fast growth worldwide.

In the folk medicine, *B. pilosa* has been used with variable indications between countries. *B. pilosa* herb was acceptable in Europe for its astringent, diaphoretic, and diuretic effects [3]. In traditional Chinese medicine, *B. pilosa* is used to manage diabetes, inflammation, dysentery, and pharyngitis [4]. Moreover, *B. pilosa* is commonly known as Pica˜o preto in Brazil, where it is widely used for treating diabetes, ulcers, inflammation, and infections [5, 6]. In addition, the roots are regarded as applicable in treating malaria [5] and even tumors [7]. In Martinique, the herb decoction is used to manage inflammation and diabetes [1, 8]. In South Africa, the herbal tea of the aerial parts is thought to have anti-allergic and anti-inflammatory effects [9]. In Cuba, *B. pilosa* is known as an antitumor agent [6]. As dietary supplements, the dried herbs are sold in Taiwan; approximately 4 million dollars per year are marketed for diabetes management [10]. Studies of *B. pilosa* have shown its antihyperglycemic [11], antihypertensive [12], antiulcer [13], hepatoprotective [14], antipyretic [15], anti-inflammatory [9, 16], anti-leukemic [17], antimalarial [5], antibacterial [18], antioxidant [19], and antitumor effects [1, 15]. It has cytotoxic activities against several types of cancer cells [2]. Based on the reported biological activities, some countries like Brazil include *B. pilosa* as an official medicinal plant for public use [20]. The use of *B. pilosa* as a medicinal plant is feasible but further clinical trials and toxicity assessments are still rare.

The major phytoconstituents detected in *B. pilosa* are polyacetylenes [4, 11, 16], terpenes [18, 21], and flavonoids [2]. Polyacetylenes derivatives, in particular, phenylheptatriyne, are the important bioactive phytoconstituent found in *B. pilosa* essential oils [21]. Flavones, flavanones, and flavonols aglycones and/or glycosides were identified from *B. pilosa*, especially quercetin, and kaempferol glycosides [20]. Many scientific reports were proceeded on *B. pilosa*, owing to its traditional use and known bioactive constituents. Further studies are needed to highlight the action mechanism of its phytoconstituents. Hence, metabolomic profiling of *B. pilosa* is a basic step for offering undiscovered medicinal uses. In this respect, gas chromatography (GC) and high-resolution ultra-performance liquid chromatography (HR-UPLC) coupled with mass spectrometry (MS) represent prospective analytical analysis for untargeted metabolome report, providing efficient separation asides from the highly sensitive recognition of phytoconstituents. Diverse kinase enzymes activate transcription factors that transcribe numerous carcinogenic markers; consequently, kinase inhibitors may rationalize the antiproliferative activity [22]. Hence, the current study aimed to execute chemical profiling, in vitro antiproliferative assessment of *B. pilosa*, and verification with computational study on kinase and aldose reductase enzymes.

## Material and Methods

### Plant Material and Extraction

*B. Pilosa* L. was collected from Beni-Sueif, Egypt in May 2022. The plant identity was performed in the Faculty of Science, Beni-Sueif University, Beni-Sueif, Egypt. The plant aerial part was washed with tap water, dried under shade (20 days), and ground into a fine powder. The powdered materials (1 kg) were extracted in Soxhlet with aqueous ethanol (70%, 4 × 2 L, 2 h) and then filtered. The filtrate was evaporated (Rotavapor®, BÜCHI, Switzerland) [23].

### In Vitro Antiproliferative Assay

The American Type Culture Collection (Manassas, VA, USA) provided SNB-19 (brain cancer cells) and SK-MEL-5 (skin cancer cells). Cell lines were cultured and maintained in RPMI-1640 medium with fetal bovine serum and penicillin/streptomycin (10% and 1%, respectively). The antiproliferation assay of *B. Pilosa* extract was proceeded by MTT assay [24]. Serial dilutions (100–1.56 mg mL^−1^) of *B. Pilosa* extract in DMSO were added to the cells (1 × 10^3^ cells mL^−1^) which were placed in 96-well plates. Furthermore, MTT (10 µL) was added to each well and incubated at 37 °C for 4 h. Using a microplate ELISA reader (FLUO star Omega, Labtech, Germany), the absorbance (Abs) of the produced formazan was measured (490 nm). Each concentration was examined in triplicates and the statistical analysis was done using GraphPad Prism 6 (La Jolla, CA, USA). The cell viability % = [Abs of tested cells/ Abs of control cells)] × 100. The values of cell viability % were plotted versus the concentrations using non-linear regression analysis of Sigmoidal dose–response curve [25].

### GC–MS Analysis

The lipoidal composition of *B. pilosa* was analyzed using a Trace GC-TSQ-MS (Thermo Scientific, TX, USA) over a capillary column TG-5MS. The column temperature was set at 50 °C (2 min), followed elevated by 5 °C/min to 250 °C and then by 30 °C/min to 300 °C (2 min). The injector and MS transfer line were maintained at 270 and 260 °C, respectively. Samples (1 µl) were injected and carried on Helium (1 mL/min) using an Autosampler AS1300 connected to a GC in split mode. Mass spectra covering *m/z* 50–650 were obtained and furthermore were compared with databases: NIST 14 and WILEY 09 [26].

### HR-UPLC/T-TOF–MS/MS Analysis

The *B. pilosa* extract was analyzed at the Proteomics and Metabolomics unit of the Children’s Cancer Hospital, Cairo, Egypt. An Exion LC Triple TOF 5600 + system (SCIEX, Framingham, MA, USA) equipped with an X select HSS T3 C_18_ column (Waters Corporation, CT, USA) was used in negative and positive modes. Extract (50 mg) was dissolved in a working solvent: MilliQ water:methanol:acetonitrile (50:25:25, 50 µL) and then was diluted with the working solvent [23]. Samples (1 µg/µL, 10 µL) were injected using the following: solvent A was ammonium formate buffer (5 mM, pH 8) containing 1% methanol, for the negative mode, while in the positive mode, solvent A was ammonium formate buffer (5 mM, pH 3) containing 1% methanol, and in both modes, solvent B was 100% acetonitrile. The gradient elution (0.3 mL/min) was performed as follows: isocratic 90%:10% (0–1 min), linear from 90%:10% to 10% and 90% (1.1–20.9 min), isocratic 10%:90% (21–25 min), and finally isocratic 90% and 10% (25.1–28 min) of solvents A and B, respectively. The metabolites were recorded by Analyst TF 1.7.1, Peak view 2.2 (SCIEX, Framingham, MA, USA), and MS-DIAL 3.70 softwares [23]. MS (50–1100 *m/z*) was done on a Triple TOF 5600 + system equipped with a Duo-Spray source operating in the electron spray ionization mode (AB SCIEX, Framingham, MA, USA). The metabolites were characterized by generating the formula with an error limit of 20 ppm and considering *Rt*, MS2 data, compared to databases and literature [27].

### Molecular Docking Study

MOE software (version 2016.10, Chemical Computing Group Inc., Montreal, Canada) was used. The structures of aldose reductase (AKR1B1; PDB: 2IKI) and ribosomal S6 kinase (RSK2 kinase; PDB: 3UBD) were obtained from the RCSB protein data bank. The standard preparation of target protein was applied. The target was validated by redocking the co-crystallized ligands (IDD388 and SL0101 to AKR1B1 and RSK2, respectively) offering low binding energy score (*S*) and small RMSD value. The essential amino acids were defined. The validated target was used to predict metabolite-target interactions [28].

## Results and Discussion

### In Vitro Antiproliferative Assay

Recent studies informed that *B. pilosa* has a promising in vitro and in vivo anticancer activity [29, 17]. However, its action mechanism has not been fully understood. The antiproliferation activity was evaluated for *B. pilosa* ethanol extract on SNB-19 and SK-MEL-5. The *B. pilosa* extract produced a decrease in cell viability of SNB-19 and SK-MEL-5 cells (IC_50_ 1.66 ± 0.06 and 4.04 ± 0.14 mg/mL, respectively) comparable to doxorubicin (IC_50_ 2.42 ± 0.08 and 11.82 ± 0.41 µg/mL, respectively).

### GC–MS Analysis of the Lipoidal Matter

Quantitation was based on separated compounds’ relative peak area, *Rt*, and their area percentages. The GC–MS chromatogram revealed the detection of 19 major metabolites (66.59%) (Table 1, Fig. 1). The percentage of identified unsaturated FAs (11.38%) was higher than the percentage of saturated FAs (8.69%). At the same time, sterols were found to be higher than hydrocarbons percent (39.92% and 6.6%, respectively). Oleic acid is the major unsaturated FA (6.14%). However, palmitic and stearic acids are the major saturated FAs (6.14% and 2.55%, respectively). The percentage of identified sterols was higher than that of hydrocarbons (39.92% and 6.6%, respectively). Lupene-3,28-diol/botulin is the major sterol, followed by sitostenone (24.95% and 6.17%, respectively) [30].

**Table 1:** Identified metabolites detected in *B. pilosa* lipoidal matter using GC–MS analysis

#	*Rt*	Name	Formula	M.Wt	Area %
1	26.20	Palmitic acid, methyl ester	C_17_H_34_O_2_	270	2.55%
2	26.96	Palmitic acid	C_16_H_32_O_2_	256	3.59%
3	29.33	Linoleic acid, methyl ester	C_19_H_34_O_2_	294	1.32%
4	29.47	Oleic acid, methyl ester	C_19_H_36_O_2_	296	4.45%
5	29.66	Tetramethyl-hexadecenol	C_20_H_40_O	296	1.14%
6	29.66	Methyl-octadecadienol	C_19_H_36_O	280	1.14%
7	29.99	Stearic acid, methyl ester	C_19_H_38_O_2_	298	1.11%
8	30.22	Oleic acid	C_18_H_34_O_2_	282	5.03%
9	30.67	Stearic acid	C_18_H_36_O_2_	284	1.44%
10	32.77	Stigmasterol	C_29_H_48_O	412	3.34%
11	38.41	Stigmastanol	C_29_H_52_O	416	0.67%
12	38.89	Heptatriacotanol	C_37_H_76_O	536	1.03%
13	39.68	Stigmastadiene-3-one	C_29_H_46_O	410	3.23%
14	39.97	lupene-3,28-diol/betulin	C_30_H_50_O_2_	442	24.95%
15	40.65	Linoleic acid ethyl ester	C_20_H_36_O_2_	308	0.58%
16	41.06	Sitostenone	C_29_H_48_O	412	6.17%
17	41.28	Cyclolanostan-3-ol, acetate	C_32_H_54_O_2_	470	1.56%
18	41.63	Tocopherol	C_29_H_50_O_2_	430	1.48%
19	42.68	Ethyl iso-allocholate	C_26_H_44_O_5_	436	1.81%
		% Identified saturated fatty acids			8.69%
		% Identified unsaturated fatty acids			11.38%
		% Identified sterols			39.92%
		% Identified hydrocarbons			6.6%
		% of total identified compounds			66.59%

**Fig. 1:**
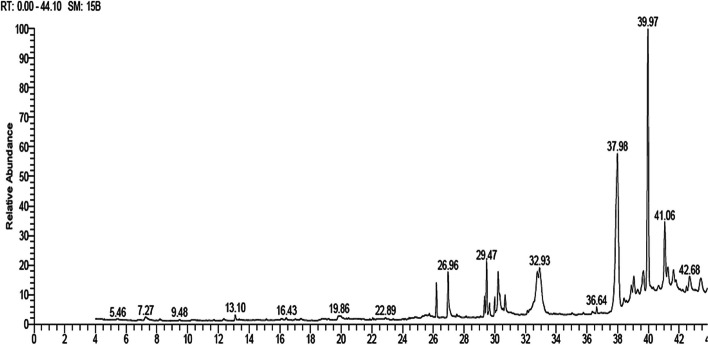
GC–MS chromatogram of *B. pilosa* lipoidal matter

### UPLC/T-TOF–MS/MS Analysis

LC–MS can analyze a wide range of metabolites; they offer tools for dissecting immense plant biodiversity. The metabolomic profile of *B. pilosa* was studied using ESI–MS/MS in positive and negative modes. Flavones, flavanones, and flavonols aglycones with their glycosides or glucuronic derivatives were identified, with a high abundance of quercetin derivatives, alongside phenolic and organic acids (Table 2, Fig. 2). The major content of flavonoids reflects the plant’s diverse biological activities.

**Table 2:** Identified metabolites in *Bidens pilosa* extract via UPLC-MS/MS using negative and positive ionization modes

#	R*t*	Metabolites	Formula	[M-H]^−^	[M + H]^+^	Error PPM	MS^2^
		**Flavonoids**					
1	4.80	Hesperidin	C_28_H_34_O_15_	609.1324		0.3	463, 301, 177, 151
2	4.86	Kaempferol-*O*-hexuronide	C_21_H_18_O_12_	461.077		0.1	285, 257, 135
3	5.09	Quercetin-*O*-hexuronide	C_21_H_18_O_13_	477.0676	479.1381	− 6.6	301, 179, 151
4	5.33	Luteolin-di-*O*-hexoside	C_27_H_30_O_16_	609.1437	611.18	8.6	447, 285, 151
5	5.38	Luteolin-*C*-hexoside	C_21_H_20_O_11_	447.0947		− 2.9	357, 327, 285, 151, 135
6	5.52	Baicalein-*O*-hexuronide	C_21_H_18_O_11_	445.077	447.1028	2.2	269, 117
7	5.64	Quercetin-*O*-di-hexoside	C_27_H_30_O_17_	625.1737	627.2105	6.1	463, 301, 283
8	6.18	Isoquercitrin	C_21_H_20_O_12_	463.0918		0.5	301, 283,255,151
9	6.41	Apigenin-*C*-hexoside	C_21_H_20_O_10_		433.1357	0.2	343, 313, 271
10	6.48	Rutin	C_27_H_30_O_16_		611.1806	2.2	465, 303
11	6.50	Eriodictyol-*O*-hexoside	C_21_H_22_O_11_	449.1194		0.7	287, 151, 135
12	6.52	Hyperoside	C_21_H_20_O_12_	463.0938	465.1223	0.8	301, 271, 255, 151
13	6.57	Apigenin-*O*-hexoside	C_21_H_20_O_10_	431.0989	433.1263	− 9.5	269,
14	6.59	Maritimetin-*O*-hexoside	C_21_H_20_O_11_	447.0909	449.1236	4.2	285
15	6.59	Kaempferol-*O*-neohesperidoside	C_27_H_30_O_15_	593.1467		0.3	285
16	6.62	Kaempferol-*O*-bis-deoxyhexoside	C_27_H_30_O_14_	577.1507	579.1816	6.7	431, 285
17	6.66	Luteolin-*O*-hexoside	C_21_H_20_O_11_	447.0921		2.7	285
18	6.66	Isorhamnetin-*O*-deoxyhexosyl-hexoside	C_28_H_32_O_16_	623.159	625.1852	− 0.2	315, 300
19	6.79	Okanin-*O*-hexoside	C_21_H_22_O_11_		451.1404	− 0.7	289, 271, 179, 163, 153
20	6.91	Naringenin-*O*-hexoside	C_21_H_22_O_10_	433.1115	435.1358	4.5	271, 151, 119
21	7.10	Vitexin-*O*-deoxyhexoside	C_27_H_30_O_14_	577.1575		− 3.8	431, 269
22	7.16	Gossypin	C_21_H_20_O_13_	479.1038		3.8	317
23	7.27	Syringetin-*O*-hexoside	C_23_H_24_O_13_	507.1129	509.1445	− 15.4	492, 345
24	7.30	Quercetin-*O*-hexoside	C_21_H_20_O_12_	463.1217	465.1522	0.4	301, 283, 135
25	7.46	Kaempferol-*O*-pentoside	C_20_H_18_O_10_		419.1226	− 2.5	287, 259, 231
26	7.46	Quercetin-*O*-hexosyl-pentoside	C_26_H_28_O_16_		597.1898	− 10.6	435, 303
27	7.54	Isorhamnetin-*O*-hexoside	C_22_H_22_O_12_	477.1333		4.9	315, 300, 151
28	7.89	Phlorizin	C_21_H_24_O_10_	435.1261		6.6	273, 151, 119
29	8.63	Quercetin-*O*-pentoside	C_20_H_18_O_11_	433.1016	435.0974	8.7	301, 193, 161, 151,
30	8.66	Eriodictyol	C_15_H_12_O_6_	287.0629	289.0796	0.5	213, 151, 135, 107
31	8.83	Acacetin-*O*-rutinoside	C_28_H_32_O_14_	591.1729		− 1.2	283, 268
32	8.95	Kaempferol-*O*-(p-coumaroyl)-hexoside	C_30_H_26_O_13_	593.1296		− 0.5	447, 285
33	9.22	Quercetin	C_15_H_10_O_7_	301.0233	303.0576	1.5	255, 193, 151, 135, 121
34	10.13	Apigenin	C_15_H_10_O_5_	269.0447	271.0641	− 3.2	159, 151, 133, 117
35	10.31	Hesperetin	C_16_H_14_O_6_	301.0724	303.0969	− 0.4	286, 151, 134
36	10.34	Luteolin	C_15_H_10_O_6_	285.0397	287.0589	3.3	257, 177, 151, 133, 107
37	10.56	Trihydroxy-methoxyflavone	C_16_H_12_O_6_	299.0556		− 1.7	284, 256, 151
38	13.16	Acacetin	C_16_H_12_O_5_	283.0606	285.0812	0.4	268, 151, 131
39	13.43	Isorhamnetin	C_16_H_12_O_7_	315.0881	317.076	6.8	300, 269, 151, 107
40	14.35	Naringenin	C_15_H_12_O_5_	271.0979	273.086	12.4	225, 136, 122
41	19.81	Rhamnetin	C_16_H_12_O_7_		317.122	6.1	299
42	19.82	Kaempferide	C_16_H_12_O_6_		301.1492	0.8	
		**Phenolic acids**					
43	1.24	Gentisic acid	C_7_H_6_O_4_	153.0193		0.8	109, 91
44	1.24	Caffeic acid	C_9_H_8_O_4_	179.055		− 0.6	161, 135,
45	1.73	Chlorogenic acid	C_16_H_18_O_9_	353.0866	355.1069	3.1	191, 179, 161, 135
46	2.52	Homogenentisic acid	C_8_H_8_O_4_	167.0331		0.5	149, 123, 108
47	2.77	Hydroxybenzoic acid	C_7_H_6_O_3_	137.0242		4.4	93, 75
48	3.37	Coumaric acid	C_9_H_8_O_3_	163.0233	165.093	10.2	119, 101
49	4.26	Protocatechuic acid	C_7_H_6_O_4_	153.0183		− 4.3	135, 109, 91
50	4.82	Dihydroxymandelate	C_8_H_8_O_5_	183.0079	184.9872	24.8	139, 109
51	5.24	Sinapic acid-*O*-hexoside	C_17_H_22_O_10_	385.1803		8.4	223, 205
52	6.50	Methoxysalicylic acid	C_8_H_8_O_4_	167.0341		2	152, 123, 108
53	8.40	Ferulic acid	C_10_H_10_O_4_	193.0501	195.1168	1.4	178, 161, 149, 133
		**Coumarin**					
54	1.27	Hydroxy-Methylcoumarin	C_10_H_8_O_3_	175.0423		7.9	157, 131, 113
55	2.68	Esculin	C_15_H_16_O_9_	339.0741	341.0936	− 3	177, 133
56	4.68	Dihydroxycoumarin	C_9_H_6_O_4_	177.0187	179.0312	0.9	149, 133, 105
57	7.09	Scopoletin	C_10_H_8_O_4_		193.0547	− 7.1	178
		**Acids**					
58	1.01	Malic acid	C_4_H_6_O_5_	133.0145		− 0.9	115, 89, 71
59	1.04	Maleic acid	C_4_H_4_O_4_	115.0022		9.3	71
60	1.06	Hydroxy-butyric acid	C_4_H_8_O_3_	103.002		9.4	59
61	1.07	Lactic acid	C_3_H_6_O_3_	89.02383		− 0.6	71
62	1.11	Succinic acid	C_4_H_6_O_4_	117.0177		9	99, 73
63	1.15	Citrate	C_6_H_8_O_7_	191.0556		− 0.3	173, 129, 111, 85
64	1.19	Tartrate	C_4_H_6_O_6_	149.0448		3.2	131, 89, 87
65	1.25	Citramalate	C_5_H_8_O_5_	146.9487		3.6	129, 85
66	1.28	Isopropylmalic acid	C_7_H_12_O_5_	175.0605		− 1	157, 131, 113, 69
67	1.35	Methylglutaric acid	C_6_H_10_O_4_	145.0258		13.1	127, 109, 101
68	2.27	Phenyllactic acid	C_9_H_10_O_3_	165.0562		1	147, 121, 103
69	4.90	Quinic acid	C_7_H_12_O_6_	191.0559		0.9	173, 127, 93
70	5.09	Shikimic acid	C_7_H_10_O_5_	173.0432		− 4.3	155, 137, 131, 111, 93
		**Amino acids**					
71	1.11	Arginine	C_6_H_14_N_4_O_2_		175.121	− 4.3	
72	1.55	Oxoproline	C_5_H_7_NO_3_		130.0488	3.9	
73	1.71	Hydroxyproline	C_5_H_9_NO_3_	130.0862		4.2	
74	2.15	Phenylalanine	C_9_H_11_NO_2_		166.0876	− 0.6	
75	2.80	Tryptophan	C_11_H_12_N_2_O_2_	203.0821		1.3	
76	3.94	Homoisoleucine	C_7_H_15_NO_2_	144.0459		0.8	
		**Fatty acids**					
77	12.82	Hydroxy-hexadecanoic acid	C_16_H_32_O_3_	271.2264		9.1	253, 212
78	18.48	Linolenic acid	C_18_H_30_O_2_	277.2177		0.5	233
		**Sugar derivatives**					
79	1.36	Mannitol	C_6_H_14_O_6_	181.0724		− 0.8	
80	2.23	Maltitol	C_12_H_24_O_11_	343.1402		− 0.5	
81	2.46	Maltotriose	C_18_H_32_O_16_	503.1371		3.8	341, 179
82	3.82	Melibiose	C_12_H_22_O_11_	341.0872		7.6

**Fig. 2:**
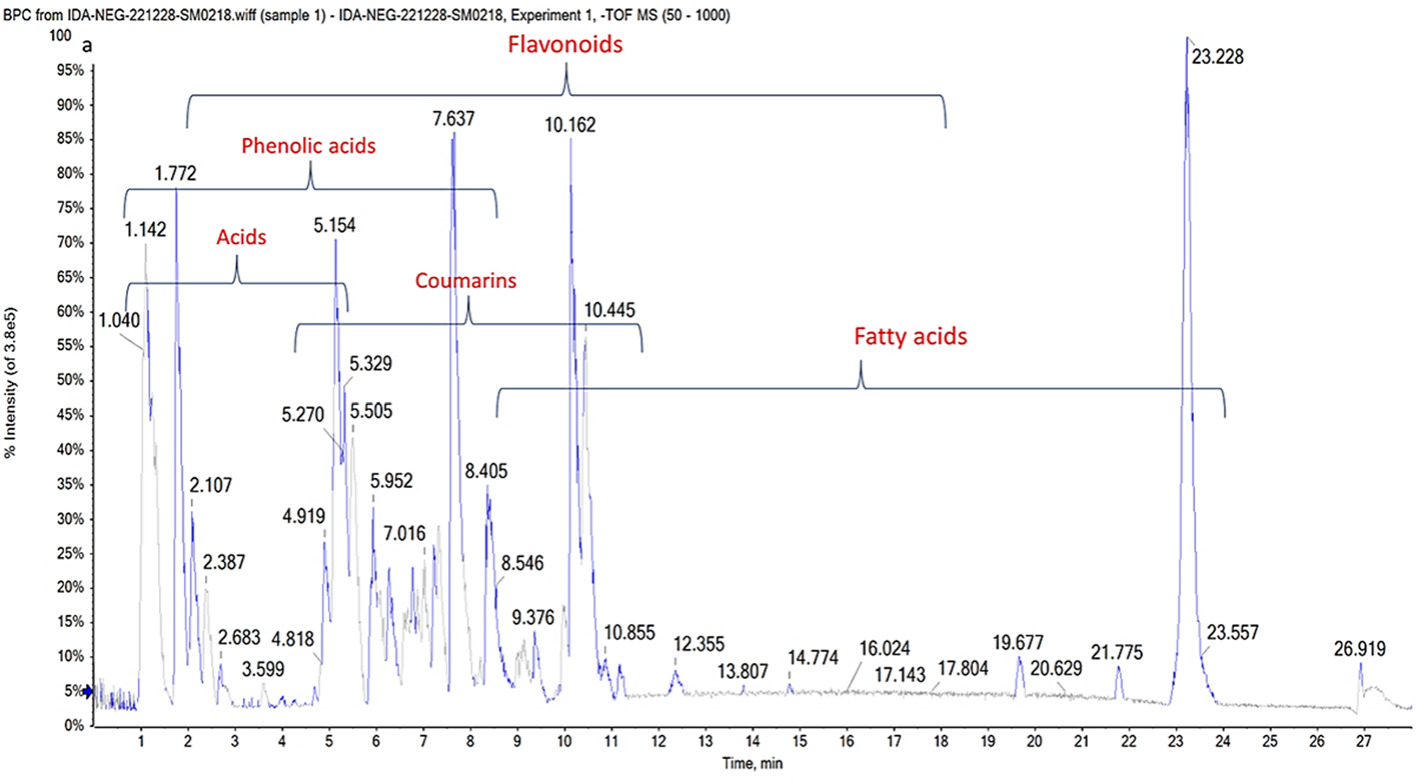
Base peak chromatogram obtained from UPLC/T-TOF–MS/MS analysis of *B. pilosa* extract in negative ionization mode

### Flavone Identification

**Fig. 3:**
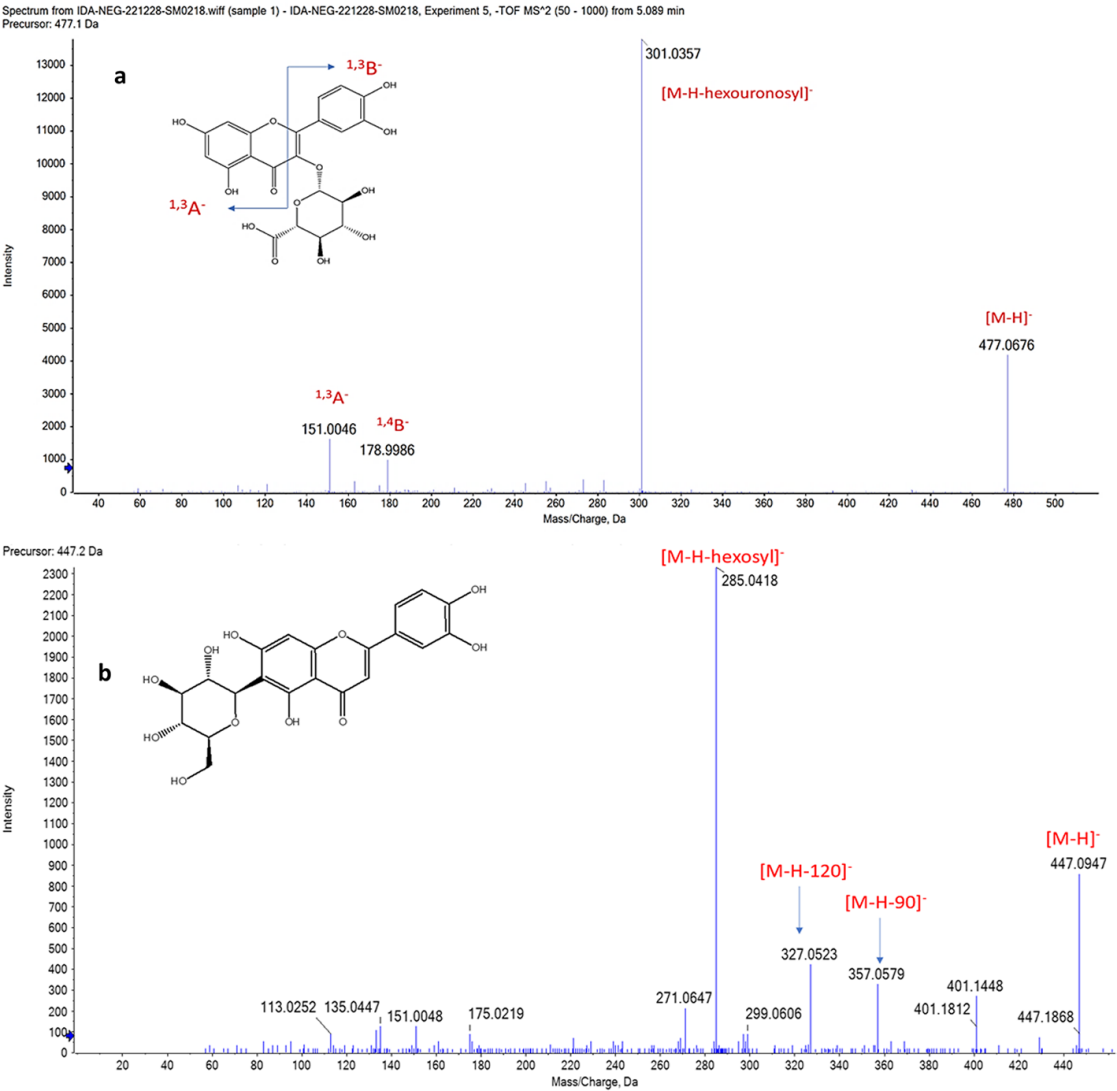
Mass fragments of major classes identified from *Bidens pilosa* extract: **a** quercetin-*O*-hexuronide (3) and **b** luteolin-*C*-hexoside (5)

Hesperidin (1) showed a deprotonated molecular ion peak at *m/z* 609.1324 and a subsequent loss of 146 and 308 Da, indicating the loss of deoxyhexosyl and deoxyhexosyl-hexosyl moieties yielding fragments at *m/z* 463 and 301 (hesperitin aglycone), respectively, with the characteristic fragments for flavonoids (177 and 151). Moreover, hesperetin (35) showed a deprotonated molecule at *m/z* 301.0724, besides the fragments of hesperetin aglycone (*m/z* 151,134). Luteolin-di-*O*-hexoside (4) showed a deprotonated molecule at *m/z* 609.1437 and subsequent losses of 162 and 324 Da at *m/z* 447 and 285 of hexosyl and di-hexosyl moieties, respectively. Luteolin-*C*-hexoside (5) showed a deprotonated molecule at *m/z* 447.0947 and the daughter peaks at *m/z* 357 and 327 representing [M-H-90]^−^ and [M-H-120]^−^, respectively, indicating C-linkage glycoside; the luteolin aglycone moiety was confirmed at *m/z* 285 (Fig. 3). Luteolin-*O*-hexoside (17) showed a deprotonated signal at *m/z* 447.0921 and a neutral loss of hexosyl moiety at *m/z* 285. The molecular ion peak [M-H]^−^ of luteolin (36) was noticed at *m/z* 285.0397, besides the characteristic fragments of luteolin aglycone (*m/z* 257, 177, 151, 133, and 107) (Table 2) [23].

Apigenin-*C*-hexoside (9) displayed a protonated molecule at *m/z* 433.1357 and the characteristic peak at *m/z* 313 [M + H-120]^+^ indicating C-linkage hexoside; moreover, base peak signal at *m/z* 271 [M + H-hexosyl]^+^ signifies apigenin. The MS2 spectrum of the deprotonated molecule at* m/z* 431.0989 showed a daughter peak at *m/z* 269 [M-H-sugar]^−^, with the major fragments of apigenin, which was identified as apigenin-*O*-hexoside (13). The MS2 spectrum of the deprotonated molecule at* m/z* 269.0447 showed the loss of B-ring-H_2_O moiety yielding the daughter ion at *m/z* 159, besides the fragments at *m/z* 151, 133, and 117 characteristics for apigenin (34) (Table 2 ) [23].

Baicalein-*O*-hexuronide (6) showed a deprotonated molecule at *m/z* 445.0770 and the loss of 176 amu equivalent to hexuronyl moiety resulting in the base peak at *m/z* 269, representing baicalein aglycone. The MS2 spectrum of a deprotonated molecule at *m/z* 577.1575 showed two subsequent losses of 146 and 308 amu, representing the deoxyhexosyl and deoxyhexosyl-hexosyl moieties yielding the product ions at *m/z* 431 and 269, respectively, with the typical fragmentation pattern of vitexin, which was proposed as vitexin-*O*-deoxyhexoside (21). The deprotonated molecular ion peak of acacetin-*O*-rutinoside (31) was observed at *m/z* 591.1729 and the daughter ion at *m/z* 283, representing losses of 308 amu (rutinosyl moiety). Acacetin (38) showed a deprotonated molecule at *m/z* 283.0606, besides signals at *m/z* 151 and 131 representing ^1,3^A^−^ and ^1,3^B^−^, respectively, for acacetin aglycone (Table 2 ) [27].

### Flavonol Identification

Quercetin-*O*-hexuronide (3) was detected by its parent ion at *m/z* 477.0676 [M-H]^−^ and a fragment at *m/z* 301 for losing of hexuronyl moiety (176 Da), besides the characteristic fragments of quercetin aglycone *m/z* 179 and 151 for ^1,4^B^**−**^ and ^1,3^A^**−**^, respectively (Fig. 3). The MS2 spectrum of the deprotonated molecule at *m/z* 625.1737 showed two subsequent losses at *m/z* 463 and 301 representing [M-H-hexosyl]^**−**^ and [M-H-di-hexosyl]^**−**^, respectively, moreover another fragment at *m/z* 283 [Ag-H_2_O]^**−**^ with the major fragments of quercetin, which was proposed as quercetin-*O*-di-hexoside (7). Isoquercitrin (8) was detected by its parent ion at *m/z* 463.0918 [M-H]^**−**^ and a fragment at *m/z* 301 [M-H-sugar]^**−**^, moreover, characteristic flavonoid fragments at *m/z* 283, 255, and 151. Rutin (10) was identified by its protonated molecule at *m/z* 611.1806. Moreover, deoxyhexosyl and deoxyhexosyl-hexosyl moieties were confirmed by the two daughter ions at *m/z* 465 and 303, respectively. Hyperoside (12) showed a deprotonated molecule at *m/z* 463.0938 and a loss of hexosyl at *m/z* 301 with ^1,3^A^**−**^ fragment which signifies quercetin aglycone. Quercetin-*O*-hexoside (23) was identified by its deprotonated molecule at *m/z* 463.1217; moreover, hexosyl and Ag-H_2_O moieties were confirmed by the two daughter ions at *m/z* 301 and 283, respectively, with another fragment at *m/z* 135 representing ^0,2^A^**−**^. Quercetin-*O*-hexosyl-pentoside (26) was identified by its protonated molecule at *m/z* 597.1898; moreover, hexosyl and hexosyl-pentosyl moieties were confirmed by two daughter ions at *m/z* 435 and 303, respectively. The MS2 spectrum of a deprotonated molecule at *m/z* 433.1016 showed two product fragments at *m/z* 301 [M-H-pentosyl]^**−**^ and 193 [M-H-B-ring]^**−**^, besides the characteristic fragments of quercetin, which was suggested as quercetin-*O*-pentoside (29). The quercetin (33) molecular ion peak [M-H]^**−**^ was noticed at *m/z* 301.0233, besides the characteristic fragments of quercetin aglycone (*m/z* 255, 193, 151, and 135) (Table 2 ) [27].

Kaempferol-*O*-hexuronide (2) showed a deprotonated molecular ion peak at *m/z* 461.0770, and a fragment at *m/z* 285 signifies kaempferol aglycone, moreover its ^0,3^A^**−**^ fragment at *m/z* 135. Kaempferol-*O*-neohesperidoside (15) was identified by a deprotonated molecule at *m/z* 593.1467; moreover, neohesperidoside moiety was confirmed by the daughter peak at *m/z* 285. The MS2 spectrum of a deprotonated molecule at *m/z* 577.1507 showed two product ions at *m/z* 431 [M-H-deoxyhexosyl]^**−**^ and 285 [M-H-di-deoxyhexosyl]^**−**^, with the typical fragments of kaempferol, which was suggested as kaempferol-*O*-bis-deoxyhexoside (16). The molecular ion peak [M + H]^+^ of kaempferol-*O*-pentoside (25) was observed at *m/z* 419.1226, followed by daughter ions at *m/z* 287 [M + H-pentosyl]^+^, 259 [M + H-pentosyl-CO]^+^, and 231 [M + H-pentosyl-2CO]^+^. Kaempferol-*O*-(coumaroyl)-hexoside (32) showed a deprotonated molecule at *m/z* 593.1296 and a fragment at *m/z* 447 [M-H-coumaroyl]^**−**^, with another fragment at *m/z* 285 [M-H-coumaroyl-hexosyl]^**−**^ (Table 2 ) [27].

Isorhamnetin-*O*-deoxyhexosyl-hexoside (18) showed a deprotonated molecule at *m/z* 623.1590 and the aglycone fragment at *m/z* 315 [M-H-deoxyhexosyl-hexosyl]^**−**^. Isorhamnetin-*O*-hexoside (27) showed a molecule ion [M-H]^**−**^ at *m/z* 477.1333, besides the aglycone base peak at *m/z* 315. Gossypin (22) showed a deprotonated molecule at *m/z* 479.1038, and the loss of hexosyl moiety was confirmed by the peak at *m/z* 317. Syringetin-*O*-hexoside (23) showed a molecule peak [M-H]^**−**^ at *m/z* 507.1129, followed by the signal of syringetin aglycone at *m/z* 345. The protonated ion peaks [M + H]^+^ of rhamnetin and kaempferide (41, 42) were noticed at *m/z* 317.1220 and 301.1492, respectively (Table 2 ).

### Flavanone Identification

Eriodictyol-*O*-hexoside (11) showed a deprotonated molecule at *m/z* 449.1194, besides the eriodictyol aglycone fragment at *m/z* 287. Eriodictyol (30) showed a deprotonated molecule at *m/z* 287.0629, moreover, the characteristic flavonoid fragments at* m/z* 213, 151, 135, and 107. Naringenin-*O*-hexoside (20) showed a signal at *m/z* 433.1115 [M-H]^**−**^ and a loss of hexosyl moiety at *m/z* 271, besides the naringenin fragments at *m/z* 151 and 119 for ^1,3^A^**−**^ and ^1,3^B^**−**^**,** respectively. Naringenin (40) showed a signal at *m/z* 271.0979 [M-H]^**−**^, besides the fragments at *m/z* 225, 136, and 122) [30]. Maritimetin-*O*-hexoside (14) showed a signal at *m/z* 447.0909 [M-H]^**−**^ and a base peak at *m/z* 285 [M-H-hexosyl]^**−**^. In the same behavior, okanin-*O*-hexoside (19) and phlorizin (28) were identified (Table 2 ).

### Phenolic, Organic, and Amino Acid Identification

The expected losses of 18, 44, and 62 Da, equivalent to the loss of H_2_O, CO_2_, and both, respectively, were observed in MS2 fragmentations of acids. Gentisic acid (43) was detected by its deprotonated molecule at *m/z* 153.0193, moreover ions at *m/z* 109 and 91. Protocatechuic acid (49) showed a deprotonated molecule at *m/z* 153.0183 and subsequent losses of H_2_O, CO_2_, and both moieties. In the same approach, caffeic, chlorogenic, coumaric, and ferulic acids (44, 45, 48, 53) were identified. The common neutral loss of CO_2_ (44 Da) was observed in MS2 fragments of organic acids as in succinic and malic acids (58, 62) (Table 2) [27]. Various amino acids were identified such as oxoproline, hydroxyproline, and phenylalanine (Table 2).

### Molecular Docking of Quercetin and Phenolic Derivatives on Aldose Reductase Protein

**Fig. 4:**
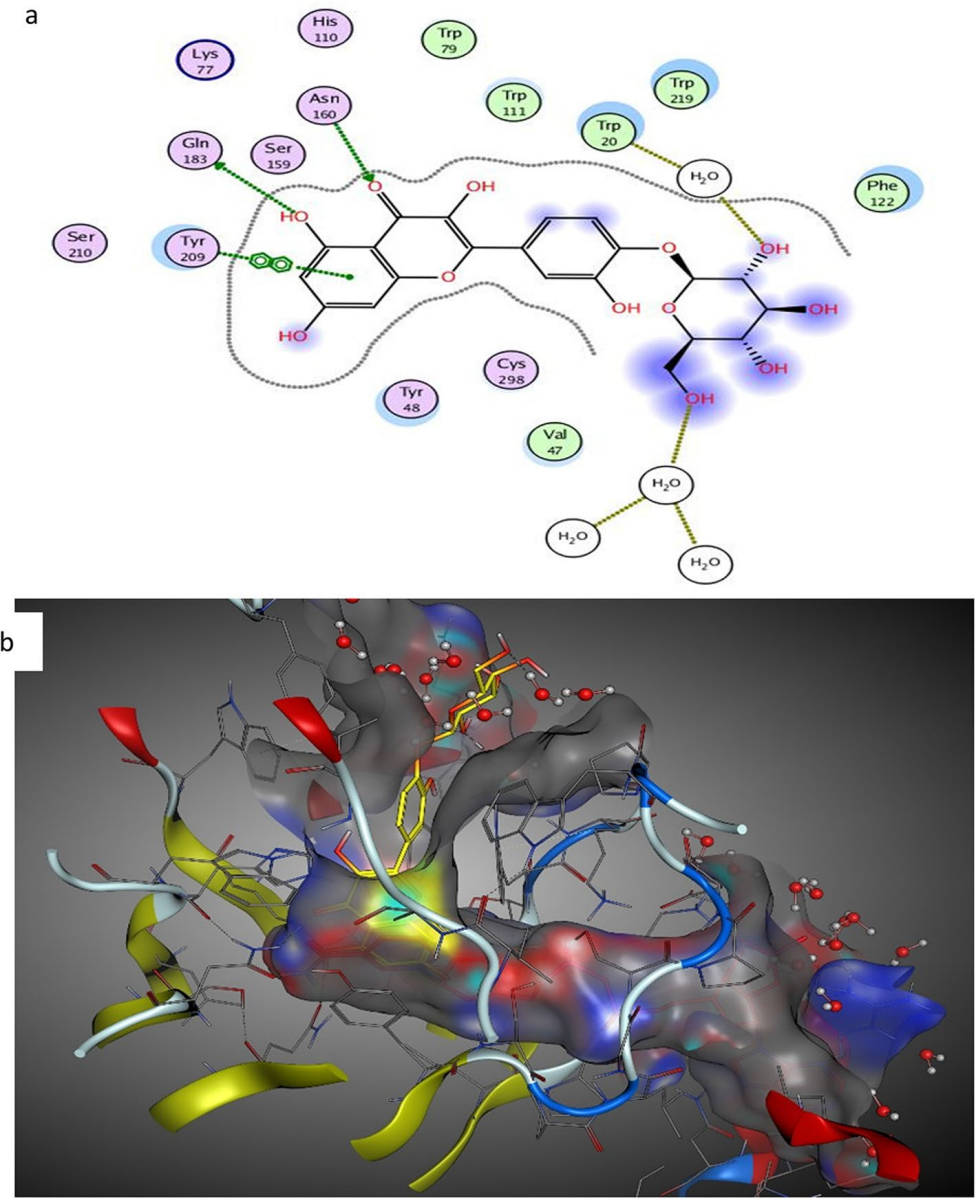
The interaction of quercetin-4′-*O*-glucoside (yellow) on aldose reductase protein (PDB ID: 2IKI) in the binding pocket: **a** 2D and **b** 3D diagrams

Inhibition of aldose reductase has a role in cancer management by preventing the activation of many transcription factors responsible for producing carcinogenic mediators [31]. The studies informed that the activity of aldose reductase increased in human cancers [32]. Inhibitors of aldose reductase showed a possible increase in the doxorubicin cytotoxic effect with a diminution in its cardiotoxicity [33, 34]. Flavonols mainly quercetin were reported as inhibitors of kinase enzymes [35, 36]. Quercetin derivatives are inhibiting tumors in rats [37]. Thus, molecular docking of *B. pilosa* metabolites on aldose reductase and kinase enzymes should be performed to rationalize its observed antiproliferative activity. The active pocket of AKR1B1 consists mainly of VAL47, TYR48, GLN49, HIS110, GLN183, and TRP111, where its co-crystalized ligand IDD388 was interacted by two H–H bonding with TYR48 and HIS110 (− 9.87 kcal mol^−1^ binding affinity). Five major metabolites were docked on aldose reductase protein with low energy of binding affinities (− 5.3 to − 7.2 kcal mol^−1^) (Table 3). Quercetin-4′-*O*-glucoside exhibited interactions (− 5.75 kcal mol^−1^ binding affinities) with four H-bonds between GLN183, HOH1218, ASN160, and HOH1056. The best docking activity was recorded on chlorogenic acid (− 7.20 kcal mol^−1^) that interacts with three H-bonds, mainly with ASP43, ILE260, and SER210 (Fig. 4).

**Table 3:** Docked conformations of quercetin and phenolic derivatives from *Bidens pilosa* on aldose reductase (AKR1B1; PDB: 2IKI) protein

Metabolites	ΔG^a^ (kcal mol^−1^)/No. of interactions	RMSD	Interactions	Distance/E (kcal/mol)
Quercetin-3-O-glucuronide	− 5.31/3	1.62	HOH1184	2.92/ − 0.8
			HOH1050	2.69/ − 0.9
			TRP20	4.73/ − 1.5
Isoquercitrin	− 5.49/3	2.32	HOH1184	3.08/ − 0.9
			HOH1050	2.88/ − 1.4
			HOH1056	3.05/ − 1.1
Quercetin-4′-O-glucoside	− 5.75/5	2.14	GLN183	3.53/ − 0.7
			HOH1218	2.92/ − 1.0
			ASN160	2.96/ − 2.5
			HOH1056	2.80/ − 1.5
			TYR209 (pi-pi)	3.69/ − 0.0
Chlorogenic acid	− 7.20/3	1.46	ASP43	2.79/ − 3.2
			ILE260	2.97/ − 4.6
			SER210	2.92/ − 2.9
Ferulic acid	− 5.3/3	1.11	HIS110	2.91/ − 1.8
			TRP20 (H-pi)	4.06 − 0.8
			TRP20 (H-pi)	4.03/ − 0.8

**Fig. 5:**
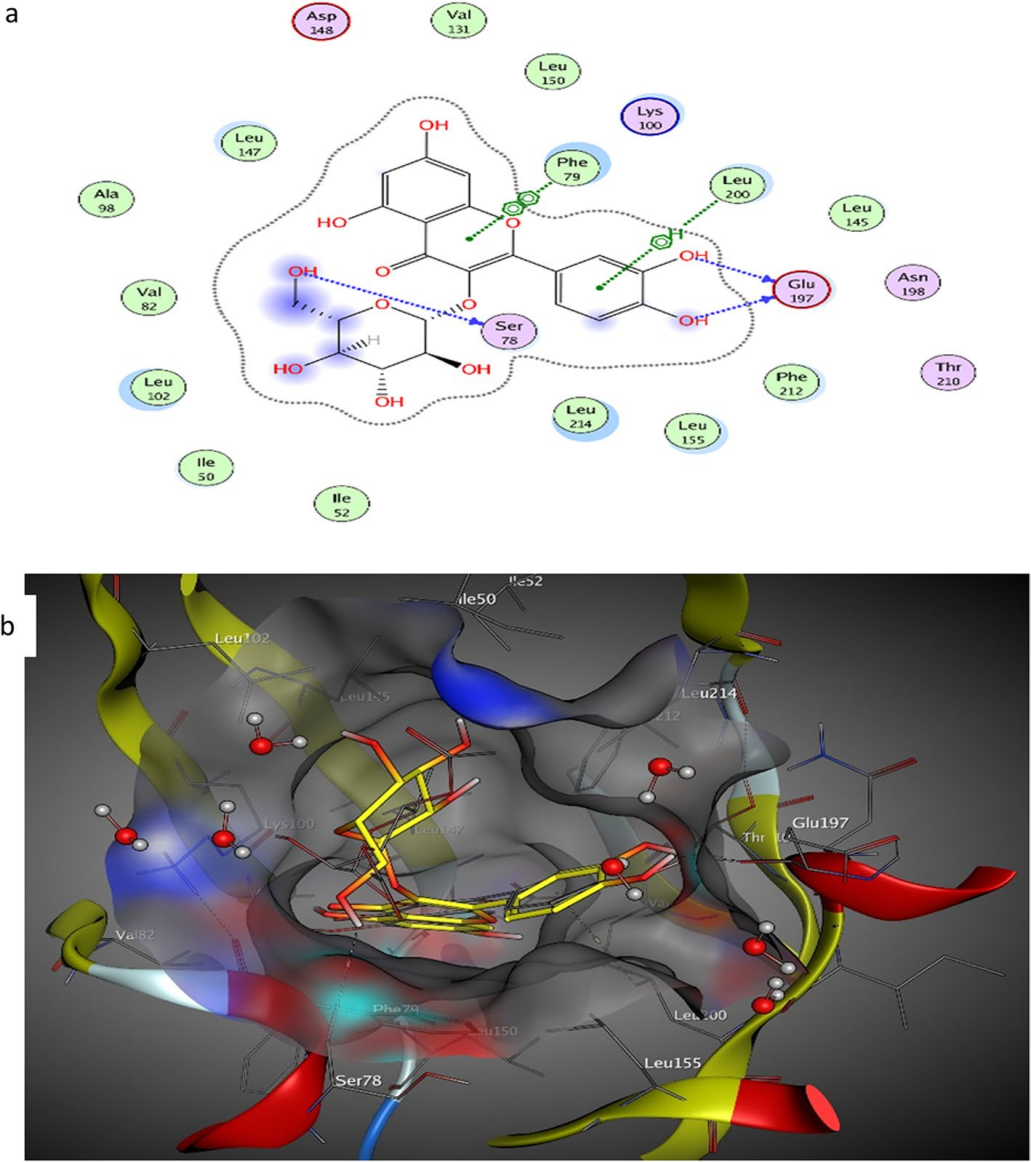
The interaction of isoquercitrin (yellow) on RSK2 protein (PDB: 3UBD) in the binding pocket: **a** 2D and **b** 3D diagrams

On the other hand, the active site of RSK2 consists mainly of PHE79, LYS100, VAL101, LYS103, LEU147, ASP148, GLU197, and LEU200 amino acids. The crystal ligand, SL0101, interacts with three H–H bonds with LYS100, ASP148, and GLU197 (− 9.54 kcal mol^−1^). The major metabolites were docked on RSK2 protein and demonstrated auspicious binding affinities (− 5.6 to − 8.89 kcal mol^−1^) (Table 4) with a variety of degrees of interactions. Isoquercitrin exhibited interactions with four H-bonds between SER78, GLU197, and LEU200 (− 8.89 kcal mol^−1^) (Fig. 5). The low binding energy of interactions between metabolites and aldose reductase and kinase proteins may justify its antiproliferative activity [38]. The phenolic and hydroxyl OH groups in metabolites are essential for interaction acting as a hydrogen bond acceptor-donator. Moreover, it was found that quercetin-4′-*O*-glucoside occupies the pocket of aldose reductase better than its co-crystallized ligand. Moreover, quercetin-4′-*O*-glucoside and isoquercitrin exhibited binding scores to targeted enzymes relatively equal to the co-crystallized ligands, expected acts as kinases and aldose reductase inhibitors leading to rationalizing its antiproliferative activity.

**Table 4:** Docked conformations of quercetin and phenolic derivatives from *Bidens pilosa* on ribosomal S6 kinase (RSK2) (PDB: 3UBD) protein

Metabolites	ΔG^a^ (kcal mol^−1^)/No. of interactions	RMSD	Interactions	Distance/E (kcal/mol)
Quercetin-3-*O*-glucuronide	− 8.29/3	1.25	GLU197	2.65/ − 3.6
			LEU200 (pi-H)	3.89/ − 1.0
			PHE79 (pi-pi)	3.85/ − 0.0
Isoquercitrin	− 8.89/5	1.41	SER78	3.18/ − 0.7
			GLU197	2.73/ − 4.3
			GLU197	2.66/ − 2.5
			LEU200 (pi-H)	3.77/ − 0.8
			PHE79 (pi-pi)	3.64/ − 0.0
Quercetin-4′-*O*-glucoside	− 7.22/3	1.10	ASP148	2.82/ − 3.4
			LYS216	2.86/ − 1.9
			PHE79 (pi-pi)	3.70/ − 0.0
Chlorogenic acid	− 6.48/3	2.76	ASP148	3.44/ − 0.8
			ASP148	2.67/ − 2.7
			LEU147 (pi-H)	3.83/ − 1.3
Ferulic acid	− 5.62/2	0.81	ASP148	2.64/ − 2.4
			LEU147 (pi-H)	4.11/ − 0.7

## Conclusion

Metabolomic profiling of the *B. pilosa* revealed its enrichment of flavonoids and phenolic acids besides coumarins, fatty, and amino acids. Moreover, *B. pilosa* extract showed in vitro antiproliferative activity. Based on in silico study, major metabolites act as inhibitors of kinases and aldose reductase leading to rationalizing its antiproliferative activity. The use of *B. pilosa* as a medicinal plant is feasible but further clinical trials and toxicity assessments are still rare.

## Data Availability

The data that support the findings of this study are available on request.
